# Is breast seroma after tumour resection associated with patient-reported breast appearance change following radiotherapy? Results from the IMPORT HIGH (CRUK/06/003) trial

**DOI:** 10.1016/j.radonc.2019.03.022

**Published:** 2019-07

**Authors:** Indrani S. Bhattacharya, Joanne S. Haviland, Carola Perotti, David Eaton, Sarah Gulliford, Emma Harris, Charlotte E. Coles, Cliona C. Kirwan, Judith M. Bliss, Anna M. Kirby, on behalf of the IMPORT Trialists

**Affiliations:** a The Institute of Cancer Research Clinical Trials and Statistics Unit (ICR-CTSU), United Kingdom; b The Royal Marsden NHS Foundation Trust, United Kingdom; c Mount Vernon Hospital, National Radiotherapy Trials QA Group, United Kingdom; d The Institute of Cancer Research, Radiotherapy and Imaging, United Kingdom; e Cambridge University, Department of Oncology, United Kingdom; f Institute of Cancer Sciences, University of Manchester, University Hospital of South Manchester, United Kingdom; g Royal Marsden NHS Foundation Trust and Institute of Cancer Research, Radiotherapy and Imaging, United Kingdom; h University College London Hospital, London, UK

**Keywords:** Breast seroma, Normal tissue effects, Patient-reported outcomes, Breast appearance change

## Abstract

**Background:**

Seroma describes a collection of serous fluid within a cavity, occurring following surgery. Seroma is associated with normal tissue effects (NTE) following breast radiotherapy, as reported by clinicians and on photographs. This study investigates the association between seroma and the NTE breast appearance change collected using patient-reported outcome measures (PROMs) in IMPORT HIGH, as well as investigating the association between breast appearance change and patient/tumour/treatment factors.

**Methods:**

Case–control methodology was used for seroma analysis within IMPORT HIGH. Cases were patients reporting moderate/marked breast appearance change and controls reported none/mild changes at year-3. One control was selected at random for each case. Seromas were graded as not visible/subtle or visible/highly visible on CT radiotherapy planning scans. Logistic regression tested associations, adjusting for patient/tumour/treatment factors.

**Results:**

1078/1149 patients consented to PROMs, of whom 836 (78%) reported whether they had 3-year breast appearance change; 231 cases and 231 controls were identified. 304/462 (66%) patients received chemotherapy. Seroma prevalence was 20% (41/202) in cases and 16% (32/205) in controls, and less frequent in patients receiving adjuvant chemotherapy [10% (24/246) compared with 29% (40/138) without]. Visible seroma was not significantly associated with breast appearance change [OR 1.38 (95%CI 0.83–2.29), *p* = 0.219]. Larger tumour size, haematoma, current smoking and body image concerns at baseline were independent risk factors.

**Conclusions:**

Seroma was not associated with patient-reported breast appearance change, but haematoma and smoking were significant risk factors. Lack of association may be related to lower prevalence of seroma compared with previous reports, perhaps reflecting patients receiving adjuvant chemotherapy in whom seroma resolves prior to radiotherapy.

## Highlights

• Seroma was not associated with patient-reported breast appearance change after breast radiotherapy.
• Haematoma and smoking were significant risk factors for patient-reported breast appearance change.
• Seroma prevalence in our study was lower than previous reports.

Seroma formation describes the collection of serous fluid within a cavity and has been reported following breast surgery. Seroma prevalence of 37% and 57% was reported in the Cambridge IMRT [1] and FAST [2] trials respectively. Seroma has been associated with increased rates of post-operative infection and haematoma, and is an independent risk factor for normal tissue effects (NTE) following radiotherapy [1].

An association between seroma and NTE has been reported in the RAPID [3] and Cambridge IMRT trials [1]. The mechanisms by which seroma may lead to NTE following radiotherapy are unknown. As well as fibrosis and retraction of the seroma cavity being possible contributing factors [4], seroma leading to larger volumes receiving radiotherapy boost doses should also be considered. In the EORTC ‘boost versus no boost’ trial there was an increased risk of fibrosis in those patients receiving a boost [5] and this risk was further increased in patients with a seroma. However, this was significant on univariate analysis only.

The majority of these trials used clinician assessments of NTE and/or serial photographs. Patient-reported outcome measures (PROMs) provide an opportunity to understand the patients’ own perception of NTE and studies have found that patients report more NTE compared with clinicians and those detected on photographs [6], [7]. However, the association between the presence of seroma and patient-reported NTE following breast radiotherapy has not been investigated to date.

This analysis from IMPORT HIGH uniquely combines comprehensive PROMs’ data with presence/absence of seroma whilst accounting for other patient, tumour and treatment factors. The primary aim of this study was to determine whether seroma is associated with patient-reported breast appearance change following breast radiotherapy. The secondary aim was to investigate associations between other patient/tumour/treatment factors and patient-reported breast appearance change.

## Methods

### Study population of IMPORT HIGH

**Fig. 1:**
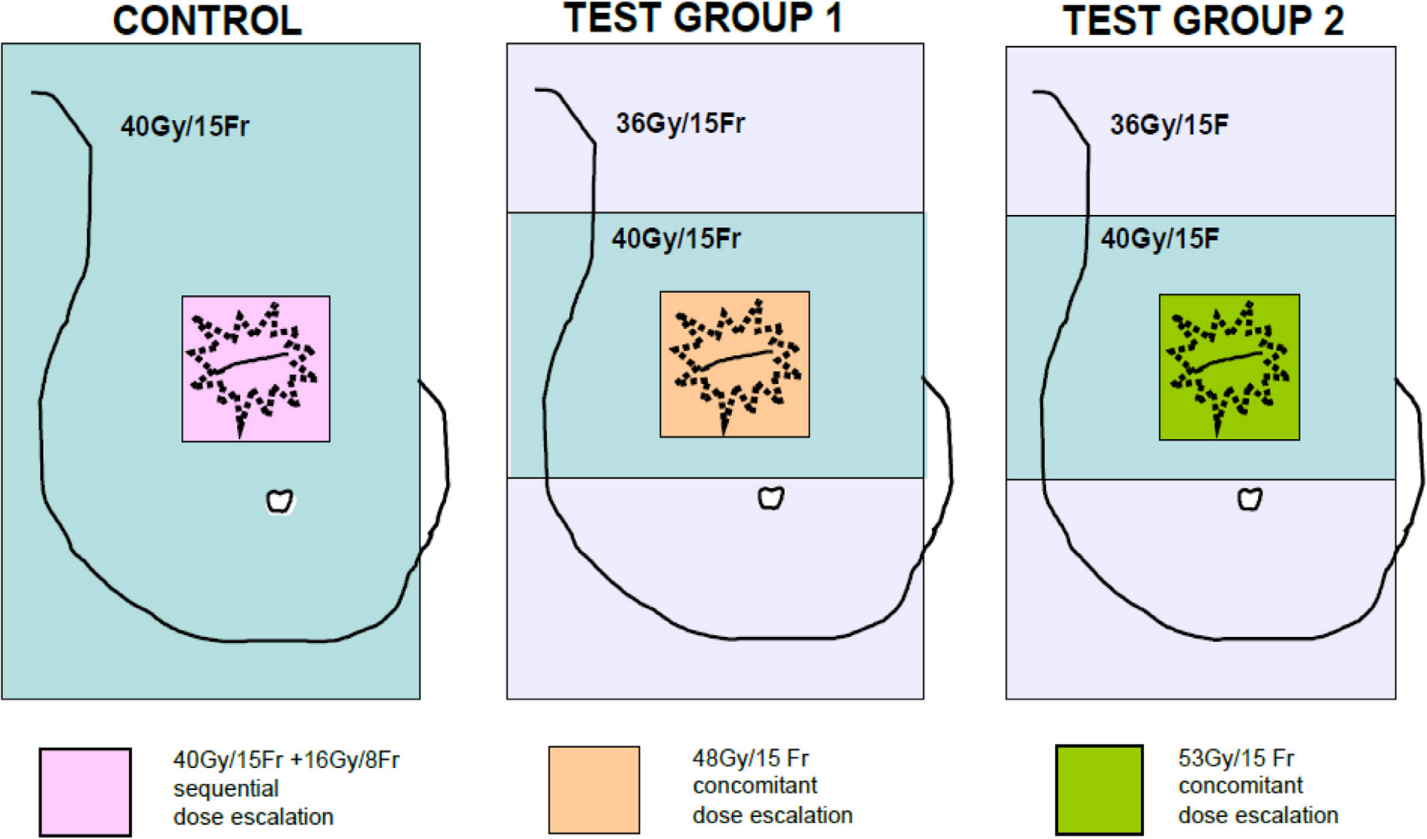
Schema of treatment groups in the IMPORT HIGH trial.

The study population consisted of patients recruited to IMPORT HIGH, a randomised, multi-centre phase III trial testing dose-escalated simultaneous integrated boost (SIB) against sequential boost each delivered by intensity-modulated radiotherapy for early-stage breast cancer with higher than average risk of local relapse. Women aged ≥ 18 after breast conservation surgery for pT1-3 pN0-pN3a M0 invasive carcinoma were eligible for IMPORT HIGH. Randomisation was 1:1:1 between 40 Gy/15 fractions (F) to whole-breast (WB) + 16 Gy/8F sequential photon boost to tumour bed (40 + 16 Gy) [control group], 36 Gy/15F to WB, 40 Gy to partial-breast + 48 Gy (48 Gy) in 15F SIB to tumour bed [test group 1] or 36 Gy/15F to WB, 40 Gy to partial-breast + 53 Gy (53 Gy) in 15F SIB to tumour bed [test group 2] (Fig. 1) [8]. The trial was initiated with a primary endpoint of breast induration at 3-years. However, this was subsequently amended to a primary endpoint of local recurrence and patient accrual extended accordingly.

IMPORT HIGH was approved by East of England Cambridge South Research Ethics Committee (08/H0305/13) and conducted in accordance with the principles of Good Clinical Practice.

### Study design – case–control methodology

For this exploratory analysis of seroma association, case–control methodology was used. As such, patients’ radiotherapy CT planning scans for seroma were reviewed in a subset of patients, rather than the whole cohort of patients in IMPORT HIGH (which would have been highly resource intensive requiring review of >2600 patients’ CT planning scans). The endpoint ‘change in breast appearance’ reported by patients at year-3 was used to define cases and controls. Patients scored breast appearance change using a 4-point scale of ‘none’, ‘a little’, ‘quite a bit’ and ‘very much’. Cases were defined as patients reporting ‘quite a bit’ or ‘very much’ (interpreted as moderate/marked breast appearance change) with controls reporting ‘none’ or ‘a little’ (interpreted as none/mild breast appearance change). The required number of controls (to equal the number of cases) was selected at random from all available controls. Cases and controls were not matched on known predictors of NTE such as breast size and surgical deficit, as these data were not available for all patients in our dataset, which would have reduced the number of cases and controls for analysis. Also, we wished to investigate associations between potential risk factors for patient-reported change in breast appearance in addition to seroma, and matching on these would have meant that we could not test them in the analyses.

### Assessment of seroma & breast density

Radiotherapy CT planning scans for cases and controls were examined for the presence of seroma. Visualisation and Organisation of Data for Cancer Analysis (VODCA v5.4, Medical Software Solutions GmbH, Hagendorn, Switzerland) software was used to view radiotherapy planning CT scans. Seroma was identified on axial CT images and graded as not visible/subtle or visible/highly visible as per methodology used in the Cambridge IMRT study [1]. Visible seroma was contoured on axial CT slices for each case using a pre-defined protocol from the Cambridge IMRT study [1] and total seroma volume recorded. Seroma contouring was undertaken by one clinical research fellow (IB) who had received training from the Chief Investigator of the Cambridge IMRT study and was blinded to patients’ case–control status.

Breast density was assessed in the contralateral breast using a ranking of 1–4 (1 = no or sparse distribution of fibroglandular tissue, 2 = small dispersed clusters of fibroglandular tissue, 3 = large cluster of fibroglandular tissue and 4 = mainly fibroglandular tissue) [2].

### Collection of dosimetric data

CT planning scan and dosimetry data were collected prospectively by the Radiotherapy Trials Quality Assurance group (RTTQA) for all IMPORT HIGH patients. Whole-breast planning target volume (PTV) dose–volume histograms (DVHs) were identified in VODCA for all cases and controls. Doses were converted into equivalent dose in 2 Gy (EQD_2_) per fraction using the Withers formula (α/β ratio 3) [9]. An α/β ratio of 3 was used following published data from the FAST and START trials, where α/β ratios were estimated at 2.3–2.6 and 3.5–4.7 respectively [10]. The whole-breast PTV mean and maximum doses (in Gray) for each patient were calculated. The tumour bed clinical target volumes (CTV) (cm^3^) were recorded on planning assessment forms (completed at the treatment centres) for all patients.

### Collection of PROM data

Within IMPORT HIGH, NTE were assessed using PROMs, photographs and annual clinician assessments. All centres were invited to participate in PROMs and photographic sub-studies (until sufficient accrual was achieved). All patients at these centres were invited to participate in the PROMs and photographic sub-studies until the required sample size for each sub-study was obtained.

PROMs were obtained at baseline, 6 months, 1 and 3 years following radiotherapy. Baseline was pre-randomisation (post-surgery, post-chemotherapy where relevant and pre-radiotherapy). PROMs collected included: Hospital Anxiety and Depression Scale (HADS) (scores of 8–10 indicating borderline anxiety or depression, and scores of 11–21 indicating case levels of anxiety or depression [11]); 10-item Body Image Scale where higher scores indicate worse body image [12] and protocol-specific questionnaire items including asking patients to score ‘change in breast appearance’ [13].

Patients consenting to the PROMs sub-study were invited to participate in the photographic sub-study which involved assessments at baseline and year-3. Breast size and surgical deficit were scored on a 3-point scale (small, medium, large) from baseline photographs by a panel of observers blinded to patient identity and treatment allocation [14]. Not all patients in the PROMs sub-study consented to photographs.

Information regarding smoking, co-morbidities (including diabetes mellitus, hypertension, cardiovascular disease, collagen vascular disease), antibiotics for tumour bed infection and haematoma were recorded at baseline. Details regarding timing of haematoma or whether the patient had any further surgical intervention for the haematoma were not recorded. Information regarding co-morbidities was collected (following a substantial amendment) 4-years after the trial opened to recruitment.

### Statistical analysis

Logistic regression was used to test associations between visible seroma and patient, tumour and treatment-related factors with moderate/marked patient-reported breast appearance change at year-3, and results summarised using odds ratios (OR, with 95% confidence intervals, CI). Each factor was initially tested in univariate analysis, and those statistically significant (*p* < 0.05) were included in a multivariable analysis.

Patient-related factors tested included age, breast size and density, smoking status, comorbidity, levels of anxiety and depression measured on HADS subscales, and Body Image Scale (BIS) score. Tumour and treatment factors tested were tumour size, grade and location, use of chemotherapy, radiotherapy treatment group, tumour bed clinical target volumes (CTV), mean and maximum dose to the whole-breast PTV, axillary lymph node status, axillary surgery, post-operative infection, haematoma, surgical deficit assessed on baseline photograph, presence of visible seroma and seroma volume. As individual dose levels were highly correlated with each other, a single dose level could not be selected. Therefore, summary metrics of mean and maximum dose were used. For analysis of seroma volume, volume was set to zero for patients without a seroma. The factors described above were clinician-reported with the exception of the PROMs (HADS subscales and BIS score).

All analyses were carried out using STATA version 14 based on a database snapshot taken on June 11 2018. The IMPORT HIGH trial is registered in the ISRCTN registry (ISRCTN47437448) and ClinicalTrials.gov (NCT00818051).

### Role of the funder

Cancer Research UK (CRUK/06/003) provided peer-reviewed approval for the IMPORT HIGH trial but had no role in this study design, data collection, data analysis, data interpretation, or writing of the report.

## Results

### IMPORT HIGH trial

IMPORT HIGH recruited 2621 patients from 77 centres. A total of 1078 of the 1149 patients from the 51 centres participating in the sub-study consented to PROMs. Year-3 questionnaires were returned by 842/1078 (78%) patients. Of these 842 patients, 836 patients provided a response for breast appearance change at year-3 and 231/836 (28%) reported moderate or marked changes (defined as cases).

### Seroma case–control analysis

In this study, 462 patients (231 cases and 231 controls) were identified (Table 1). Adjuvant chemotherapy was received by 147/231 (64%) cases and 132/231 (57%) controls, and neo-adjuvant chemotherapy by 9/231 (4%) cases and 16/231 (7%) controls. In patients who received adjuvant chemotherapy, the radiotherapy planning scan would have been done approximately 16–20 weeks post-surgery (based on standard UK practice). In patients receiving neo-adjuvant chemotherapy or no chemotherapy, the radiotherapy planning scan would be approximately 4 weeks post-surgery. Radiotherapy planning CT data were available for 407 patients (missing for 29 cases and 26 controls). Reasons for missing data included the inability to retrieve dose files from centres, corrupted dose files, or deviations from trial protocol (where patients received local standard treatment, CT planning scans and dosimetric data were not collected for these patients). There were no differences in reasons for missing data between cases and controls. Seroma prevalence was 41/202 (20%) in the cases and 32/205 (16%) in the controls. In patients receiving adjuvant chemotherapy for whom seroma data were available, 10% (24/246 patients) had seroma compared with 29% (40/138) in patients not receiving chemotherapy.

**Table 1:** Summary of univariate analyses: associations between baseline characteristics and moderate/marked change in breast appearance at 3 years in the case–control population in IMPORT HIGH.

Characteristics	Cases [Patients reporting moderate/marked change in breast appearance at 3 years] N = 231 (%)	Controls [Patients reporting none/mild change in breast appearance at 3 years] N = 231 (%)	Univariate analyses OR (95% CI)	*P* value
Age years	N = 231	N = 231	0.98 (0.96–0.996)	0.019
Median (IQR)	49 (45–52)	49 (45–57)		
*Treatment group*				
Control	84/231 (36)	77/231 (33)	1	
Test group 1	62/231 (27)	74/231 (32)	0.77 (0.49–1.21)	0.258
Test group 2	85/231 (37)	80/231 (35)	0.97 (0.63–1.50)	0.905
Tumour size (cm)	N = 231	N = 231	1.27 (1.07–1.50)	0.005
	2.1 (1.6–2.8)	1.7 (1.3–2.5)		

*Tumour grade*				
Grade 1	24/231 (10)	17/231 (7)	1	
Grade 2	99/231 (43)	93/231 (40)	0.75 (0.38–1.49)	0.418
Grade 3	108/231 (47)	121/231 (52)	0.63 (0.32–1.24)	0.182

*Lymph nodes*				
Positive	77/231 (33)	70/231 (30)	1	
Negative	154/231 (67)	161/231 (70)	0.87 (0.59–1.29)	0.485

*Tumour location*				
Central	38/230 (17)	29/230 (13)	1	
Upper outer quadrant	106/230 (46)	114/230 (50)	0.71 (0.41–1.23)	0.222
Upper inner quadrant	47/230 (20)	48/230 (21)	0.75 (0.40–1.40)	0.364
Lower outer quadrant	25/230 (11)	24/230 (10)	0.79 (0.38–1.67)	0.543
Lower inner quadrant	14/230 (6)	15/230 (7)	0.71 (0.30–1.71)	0.447
CTV boost volume in cc	N = 161	N = 166	1.02 (1.00–1.03)	0.008
Median (IQR)	15.4 (7.5–24.6)	11.6 (6.4–18.6)		

*Axillary surgery*				
No	3/231 (1)	3/231 (1)	1	
Yes	228/231 (99)	228/231 (99)	1.00 (0.20–5.0)	>0.99

*Post-op infection*				
No	189/231 (82)	207/229 (90)	1	
Yes	42/231 (18)	22/229 (10)	2.10 (1.20–3.63)	0.009

*Post-op haematoma*				
No	202/231 (87)	219/229 (96)	1	
Yes	29/231 (13)	10/229 (4)	3.14 (1.49–6.61)	0.003

*Smoking status*				
Never smoker	123/231 (53)	141/229 (62)	1	
Current smoker	41/231 (18)	21/229 (9)	2.24 (1.25–3.99)	0.006
Previous smoker	67/231 (29)	67/229 (29)	1.15 (0.76–1.74)	0.520

*Cardiovascular disease*				
No	218/229 (95)	210/226 (93)	1	
Yes	11/229 (5)	16/226 (7)	0.66 (0.30–1.46)	0.307

*Adjuvant chemotherapy*				
No	75/231 (32)	83/231 (36)	1	
Yes	156/231 (68)	148/231 (64)	1.17 (0.79–1.71)	0.433

*Baseline HADs anxiety*				
Normal (0–7)	133/214 (62)	154/218 (71)	1	
Borderline (8–10)	38/214 (18)	46/218 (21)	0.96 (0.59–1.56)	0.858
Case (11+)	43/214 (20)	18/218 (8)	2.77 (1.52–5.03)	0.001

*Baseline HADs depression*				
Normal (0–7)	167/215 (78)	184/217 (85)	1	
Borderline (8–10)	30/215 (14)	26/217 (12)	1.27 (0.72–2.24)	0.405
Case (11+)	18/215 (8)	7/217 (3)	2.83 (1.15–6.95)	0.023
Body Image Scale^*^	N = 210	N = 215	1.06 (1.03–1.09)	<0.001
Median (IQR)	9 (4–15)	5 (1–11)		

Breast Size^**^^				
Small	57/140 (41)	69/152 (45)	1	
Medium	52/140 (37)	66/152 (43)	0.95 (0.58–1.58)	0.854
Large	31/140 (22)	17/152 (11)	2.21 (1.11–4.39)	0.024

Surgical deficit^**^				
Small	86/140 (61)	119/152 (78)	1	
Medium	39/140 (28)	28/152 (18)	1.93 (1.10–3.37)	0.021
Large	15/140 (11)	5/152 (3)	4.15 (1.45–11.86)	0.008

*Seroma*				
No	161/202 (80)	173/205 (84)	1	
Yes	41/202 (20)	32/205 (16)	1.38 (0.83–2.29)	0.219
Seroma volume (cc)	N = 198	N = 203	#1.21 (1.02–1.44)	0.032
Median (IQR)^***^	20.3 (6.8–46.1)	13.6 (7.4–19.0)		

*Breast density^∼^*				
Rank 1	88/201 (44)	70/204 (34)	1	
Rank 2	51/201 (25)	57/204 (28)	0.71 (0.44–1.16)	0.175
Rank 3	50/201 (25)	51/204 (25)	0.78 (0.47–1.29)	0.330
Rank 4	12/201 (6)	26/204 (13)	0.37 (0.18–0.78)	0.009
Mean dose in Gray	N = 192	N = 197	1.08 (1.02–1.14)	0.009
	45.1 (43.2–49.2)	44.0 (42.4–48.6)		
Maximum dose in Gray	N = 192	N = 197	1.01 (0.97–1.05)	0.532
	66 (65–74)	66 (65–74)		

IQR = interquartile range ^*^Higher scores for body image scale indicate more problems (possible range 0–30). ^**^Breast size and surgical deficit scored on baseline photographs (data not available for all patients as all patients in PROMs sub-study did not participate in the photographic sub-study). ^***^For seroma volume, patients without seroma included in analysis with zero volume. ^#^Seroma volume assessed per 10 cc. ^∼^Data from 2 patients missing due to inability to assess contralateral breast and implants. Rank 1 = no or sparse distribution of fibroglandular tissue, 2 = small dispersed clusters of fibroglandular tissue, 3 = large cluster of fibroglandular tissue and 4 = mainly fibroglandular tissue. ^Breast size also assessed using whole breast PTV volume. Data for diabetes mellitus, hypertension and collagen vascular disease not shown as few patients had available data.

Statistically significant patient factors associated with 3-year moderate/marked breast appearance change in univariate analysis included younger age, larger breast size, greater breast density, current smoking, higher baseline HADS anxiety and depression scores and body image concerns at baseline. There was a large proportion of missing co-morbidity data and therefore these were not tested in univariate analysis, with the exception of cardiovascular disease.

Tumour and treatment factors associated with 3-year moderate/marked breast appearance change in univariate analysis were larger tumour size, post-operative infection, haematoma, larger surgical deficit on photographs, larger seroma volume, larger tumour bed CTV and mean dose (Table 1). There was no statistically significant association found between visible/highly visible seroma and moderate/marked breast appearance change at 3 years [OR 1.38 (0.83–2.29), *p* = 0.22]. Stratifying by adjuvant chemotherapy use, the odds ratio for the association between seroma and moderate/marked breast appearance change in patients receiving chemotherapy was 2.0 [0.82–4.86), *p* = 0.13] compared with 1.25 [(0.60–2.61), *p* = 0.55] in patients not receiving chemotherapy.

Factors which remained statistically significant in multivariable analysis were, larger tumour size, haematoma, current smoking and body image concerns at baseline (Table 2). The association between seroma volume and moderate/marked breast appearance change was no longer significant in multivariable analysis. As there was a large proportion of missing data for tumour bed CTV (135 patients missing) and also for breast size and surgical deficit assessed on photographs (170 patients data unavailable), these factors were excluded from the multivariable analysis. Whole-breast PTV recorded on CT planning scans were used in logistic regression models to represent breast size, but this was not associated with moderate/marked breast appearance change in multivariable analysis (Table 2).

**Table 2:** Summary of multivariable analyses: associations between baseline characteristics and moderate/marked change in breast appearance at 3 years.

Characteristics	Multivariable analyses Adjusted OR^*^ (95% CI)	P value
Age	0.98 (0.96–1.01)	0.243
Tumour size	1.43 (1.13–1.82)	0.003

*Post-op infection*		
No	1	
Yes	1.45 (0.68–3.07)	0.335

*Post-op haematoma*		
No	1	
Yes	5.96 (2.20–16.11)	<0.001

*Smoking status*		
Never smoker	1	
Current smoker	2.25 (1.06–4.74)	0.034
Previous smoker	1.15 (0.67–1.97)	0.613

*Baseline HADs anxiety*		
Normal (0–7)	1	
Borderline (8–10)	0.70 (0.37–1.32)	0.273
Case (11 + )	2.17 (0.97–4.87)	0.060

*Baseline HADs depression*		
Normal (0–7)	1	
Borderline (8–10)	0.90 (0.42–1.93)	0.778
Case (11+)	1.93 (0.53–6.99)	0.317
Body Image Scale	1.04 (1.00–1.09)	0.044
Whole Breast PTV volume	1.00 (0.99–1.00)	0.226
Seroma volume	1.01 (0.99–1.04)	0.209

*Breast density*		
Rank 1	1	
Rank 2	0.63 (0.34–1.16)	0.134
Rank 3	0.86 (0.44–1.68)	0.662
Rank 4	0.41 (0.16–1.08)	0.070
Mean dose to whole breast in Gray	1.05 (0.98–1.13)	0.190

^*^Odds ratios adjusted for all variables shown in the table. Rank 1 = no or sparse distribution of fibroglandular tissue, 2 = small dispersed clusters of fibroglandular tissue, 3 = large cluster of fibroglandular tissue and 4 = mainly fibroglandular tissue.

## Discussion

These results show, within IMPORT HIGH, there was no significant association between seroma and patient-reported breast appearance change at 3-years. However, haematoma, larger tumour size, current smoking and body image concerns at baseline were significant risk factors. In contrast to our findings, the Cambridge IMRT study comparing 2-dimensional radiotherapy against forward-planned IMRT using 40 Gy in 15 fractions in both treatment groups, found a significant association between seroma and inferior cosmesis on photographs at 5-years [OR = 1.8, (95%CI 1.0–3.4), *p* = 0.05] [1]. Juneja et al also showed an association between seroma and breast appearance change on photographs at 2-years [OR 3.44, (95%CI 1.28–9.21), *p* = 0.01] in the FAST-Pilot (patients received 30 Gy in 5F over 15 days) and UK FAST trials (randomising to 50 Gy in 25F versus 28.5 or 30 Gy in 5 once weekly fractions) [2].

The lack of association between seroma and patient-reported breast appearance change may be related to the low overall prevalence of seroma within the case–control study in IMPORT HIGH: 20% in the cases and 16% in the controls. Clinically, this is lower than the 37% seroma prevalence reported in the Cambridge IMRT study [1]. It is also lower than the 57% seroma prevalence reported in a case–control study using patients from the FAST-Pilot and UK FAST trials [2].

Reasons for the lower prevalence of seroma in IMPORT HIGH may be due to a larger proportion of patients receiving chemotherapy (potentially resulting in seroma resolving prior to radiotherapy) and changes in surgical practice over time. The Cambridge IMRT and FAST trials recruited between 2003 and 2007, whereas IMPORT HIGH recruited from 2009 to 2015. In the Cambridge IMRT seroma study, 122/648 (19%) patients received chemotherapy [1] compared with 304/462 (66%) patients in our case–control study in IMPORT HIGH. In the patients receiving adjuvant chemotherapy in IMPORT HIGH (with a time lag of approximately 16–20 weeks from surgery to radiotherapy planning scan), 10% (24/246 patients) had seroma compared with 29% (40/138) in patients not receiving chemotherapy. One study demonstrated that seroma volume decreases with a longer time interval from surgery to radiotherapy [15].

Chemotherapy was also considered a potential confounder in IMPORT HIGH. However, in our study, adjusting for adjuvant chemotherapy use made little difference to the estimate of association between seroma and breast appearance change. Nevertheless, seromas persisting after chemotherapy may be more stable during radiotherapy such that dosimetric heterogeneities within the tumour bed region incurred by fluctuating seroma volume will be minimised. In addition, seromas persisting following chemotherapy may maintain volume within the tumour bed such that any distortion associated with their resolution may be less likely.

Surgical practices have changed since the FAST and Cambridge IMRT trials were conducted, from leaving the excision cavity open (which may be associated with seroma formation) towards primary closure of the defect by either direct suturing of cavity walls together, local glandular mobilisation or therapeutic mammoplasty. In patients who develop a seroma in an open cavity, fibrosis and retraction of tissue surrounding the excision cavity (following seroma reabsorption) could result in a noticeable defect [4]. In contrast, there is also evidence to suggest that the seroma cavity may not always contract and new tissue may be laid down in concentric rings [16]. With increasing use of oncoplastic surgery to redistribute breast tissue into locations of volume loss particularly in those requiring extensive resections, rates of seroma are likely to have reduced. One study reported significantly lower rates of seroma in patients undergoing oncoplastic surgery compared with standard breast conserving surgery: 1.7% versus 4.4%, *p* = 0.04 [17], albeit that seromas were diagnosed clinically in this study and thus rates were lower than described in the radiotherapy literature.

It is possible that our study was underpowered to detect a moderate effect of seroma; with around 200 cases and controls the study had 78% power to detect an odds ratio of 2, based on 16% seroma prevalence in our control population (alpha = 0.05). Although there was no significant association between seroma and breast appearance change, greater seroma *volume* was associated with breast appearance change on univariate analysis. For the analysis, seroma volume was set to zero for patients without seroma. Limited patient numbers with seroma may have contributed to the lack of significance on multivariable analysis, or it may be that the association between seroma and NTE is weaker than previously reported. The RAPID trial testing partial-breast radiotherapy using 3D conformal radiotherapy versus whole-breast radiotherapy reported an association between seroma volume and adverse cosmesis at 3-years [3].

The choice of endpoint used in our case–control study may also explain our results being different to those of other published studies. PROMs provide the patient-perspective of side-effects and it has been shown that patients report a higher prevalence of NTE compared with clinicians or photographs [6], [7]. Therefore, PROMs may be a more sensitive endpoint. Furthermore, patients experiencing a large palpable seroma at baseline may be more perceptive of future NTE compared with clinicians or photographic scoring (where prior seroma may not be noted). Greater volume of seroma was associated with 3-year breast appearance change in IMPORT HIGH.

With respect to other tumour and treatment factors, haematoma was significantly associated with breast appearance change within IMPORT HIGH. Similarly, haematoma predicted moderate/severe fibrosis in the EORTC 2281-10882 ‘boost versus no boost’ trial [HR 1.80 (95%CI (1.32–2.47), *p* < 0.0001] [5]. Post-operative haematoma leading to worse cosmetic outcome may be related to glandular necrosis. Larger tumour size was also significantly associated with breast appearance change. Tumour size may be a proxy measure for surgical deficit. Larger surgical deficit at baseline predicted patient-reported breast appearance change in IMPORT LOW [18]. Also, larger excision volumes were associated with poorer cosmetic outcome in the EORTC ‘boost versus no boost’ trial [19]. With regard to patient factors, current smoking was strongly associated with patient-reported breast appearance change in IMPORT HIGH. Similarly in the RAPID trial, smoking was associated with adverse cosmesis [OR 2.42 (95%CI 1.56–3.75), *p* < 0.001] and a deterioration in cosmesis over 3-years [OR 1.58 (95%1.01–2.46), p = 0.04] [3]. Smoking has been associated with impaired wound healing, post-operative complications and increased radiation toxicity [20], [21]. Finally, body image concerns at baseline were also significantly associated with breast appearance change. Items in the BIS relate to patient perception of attractiveness and sexuality as a result of their disease or treatment. This association has not been previously investigated or reported in the literature.

### Implications of findings

We were unable to show an association between seroma and patient-reported breast appearance change, however larger tumour size, haematoma, current smoking and body image concerns at baseline were independent risk factors. This suggests that measures should be taken to reduce the risk of haematoma formation. For example, by achieving adequate haemostasis with return of patient blood pressure to normal prior to wound closure and avoidance of post-operative hypertension (eg due to pain). Also, smoking cessation should be encouraged, although we cannot determine the time interval required from smoking cessation to start of radiotherapy to reduce the risk of patient-reported breast appearance change.

In conclusion, seroma was not associated with patient-reported breast appearance change, but haematoma and smoking were significant risk factors. Lack of association may be related to lower prevalence of seroma compared with previous reports, perhaps reflecting patients receiving adjuvant chemotherapy in whom seroma resolves.

## Conflicts of interest

IB, JH, CP, DE, SG, EH, CC, CK, AK have no conflicts of interest of interest to declare. JMB discloses Research Funding: AstraZeneca, Merck Sharp & Dohme, Medivation, Puma Biotechnology, Clovis Oncology, Pfizer, Janssen-Cilag, Novartis, Roche.
